# Edentulism or Poor Oral Hygiene: Which Is the Stronger Predictor for All-Cause Mortality?

**DOI:** 10.3390/jcm14020371

**Published:** 2025-01-09

**Authors:** Sok-Ja Janket, Hasna H. Kunhipurayil, Faleh Tamimi, Markku Surakka, Huiqi Li, Thomas E. Van Dyke, Jukka H. Meurman

**Affiliations:** 1 Boston University H. M. Goldman School of Dental Medicine, Boston, MA 02118, USA; skjanket@bu.edu; 2 College of Dental Medicine, QU Health, Qatar University, Doha P.O. Box 2713, Qatar; hk1512005@qu.edu.qa; 3 Department of Oral and Maxillofacial Diseases, Kuopio University Hospital, 70200 Kuopio, Finland; markkusurakka86@gmail.com; 4 Healthy Longevity Translational Research Program, Yong Loo Lin School of Medicine, National University of Singapore, Singapore 117597, Singapore; huiqi_li@u.nus.edu; 5 ADA Forsyth Institute, Center for Clinical and Translational Research, Somerville, MA 02115, USA; tvandyke@forsyth.org; 6 Department of Oral and Maxillofacial Diseases, Helsinki University Hospital and University of Helsinki, 00290 Helsinki, Finland; jukka.meurman@helsinki.fi

**Keywords:** edentulism, oral hygiene self-care, validation of self-report, inflammation, all-cause mortality, dyslipidemia, socioeconomic status

## Abstract

**Background**: All-cause mortality consisting of several heterogeneous subgroups does not have a well-defined set of risk factors. Despite the well-described role of oral hygiene on mortality, the association between the condition of the existing dentition and mortality remains unclear. Therefore, we embarked on the current study to assess the association of oral hygiene self-care (OHS) with all-cause mortality. **Methods**: We assessed whether edentulism and the levels of OHS are associated with all-cause mortality in 476 subjects without missing values participating in the KOHH study using proportional hazard models. We designated the edentulous group as OHS0, and poor, fair, and good OHS groups as OHS1, OHS2, and OHS3, respectively. The self-reported OHS was validated against clinical measures of oral inflammation and dental cleanliness, i.e., gingival bleeding and plaque indices. We, then, compared all-cause mortality at three levels of OHS (poor, fair, good) to that of the edentulous group. To test whether the association of OHS to all-cause mortality was mediated by inflammation, we adjusted for CRP. **Results**: The validity of self-reported OHS was good demonstrating an inverse association with gingival inflammation and plaque index in a dose-response manner. The group with good OHS lived significantly longer, with a 50% lower risk of all-cause mortality. The Hazard ratio (HR) = 0.50 (95% confidence limit: 0.25–0.99), *p* = 0.045, in a model adjusted for age, smoking, body mass index, and education. Adjusting for CRP attenuated the association of OHS to all-cause mortality slightly, suggesting that this association was mediated, at least in part, by inflammation. In the final model, the poor OHS group exhibited HR = 0.98 (0.51–1.89), *p* = 0.95. The HR and *p*-value so close to 1 suggested poor OHS has a similar risk to edentulism. **Conclusions**: OHS was associated with reduced risk for all-cause mortality: the better OHS, the lower the risk for all-cause mortality. Poor oral hygiene showed a similar risk for all-cause mortality to edentulism.

## 1. Introduction

We previously reported that oral hygiene self-care (OHS) was associated with a lower risk of CVD mortality [1]. The mechanism appears to be attributable to reducing inflammation [2]. These observations raise the question of whether oral hygiene could also impact all-cause mortality. Indeed, a study that analyzed the data of the Scottish Health Survey observed significant inverse associations between the frequency of toothbrushing and markers of low-grade systemic inflammation [3]. The Scottish study participants who brushed their teeth less frequently showed elevated levels of both serum C-reactive protein (CRP) and fibrinogen. Additionally, a study conducted by Luo et al. (2022) analyzing National Health and Nutrition Examination Survey data demonstrated that significant tooth loss was more likely to have elevated CRP, and dental flossing was associated with a lower risk of elevated CRP [4]. Subsequently, a study conducted by Reichert et al. in 2015 showed that flossing and brushing of interdental spaces were associated with reduced risk for new cardiovascular events among patients with a history of CAD [5]. In another study, improved oral hygiene was associated with reduced systemic inflammatory risk factors for CVD [6].

Moreover, histopathological analysis of the atherosclerotic plaques of patients with carotid stenosis or aortic aneurysm revealed that most of the patients were edentulous (76.9%) and Streptococcus mutans was most commonly detected (100% of the time) in the atherosclerotic plaque, followed by Prevotella intermedia (7.1%) [7]. However, Porphyromonas gingivalis and Treponema denticola were not detected in the atherosclerotic plaques. This study [7] suggested the potential contribution of the cariogenic microbe, Streptococcus mutans, to atherosclerosis.

It is well established that edentulous persons have a shorter life span than dentate individuals [8]. Several studies confirmed that edentulism was associated with a higher risk of mortality [9,10,11,12], including our own [13]. However, none evaluated the condition of the remaining dentition. It is quite possible that dentate individuals may have teeth but they may be covered with dental plaque, which could result in elevated systemic inflammation.

The cause of edentulism in this cohort was due to dental caries (60%) and periodontitis (40%), respectively [14]. Since both reasons for tooth loss are the direct result of poor oral hygiene, the level of OHS could mitigate the risk of CVD as well as other inflammatory diseases affecting all-cause mortality. Therefore, the present study aims to explore the potential benefits of good oral hygiene on all-cause mortality and longevity compared with the same parameters in the edentulous subgroup.

The aims of this study were the following:

1. Is oral hygiene self-care (OHS) associated with all-cause mortality compared with the edentulous group?
2. Does inflammation mediate the pathway of oral hygiene self-care to all-cause mortality?

## 2. Materials and Methods

### 2.1. Human Subjects’ Protection

The detailed methods of this study have been previously published [15]. This study was approved by the Institutional Review Board of Kuopio University Hospital and the University of Kuopio in 1994 (IRB approval # HFO 158/94 16.9.1994 permit date November 4th, 1994). All participants provided written informed consent.

The longitudinal portion of this study where mortality data were appended to the initial case–control study was approved by the Institutional Review Board of Boston University in 2006 (IRB approval number H-24875, approved on Date of Action: June 2nd, 2006). The research was conducted in accordance with the guidelines of the Declaration of Helsinki and the Belmont Accord to safeguard the protection of human research participants.

### 2.2. Study Population

The description of the study population and the endpoint were published previously [15] but are briefly reiterated here. The Kuopio Oral Health and Heart (KOHH) study was started in 1995–1996 to explore the association between oral health and coronary artery disease (CAD). For the longitudinal part of the study, the mortality data (median follow-up of 18.8 years) were added to the baseline data to create a prospective follow-up study assessing oral infection impacts on CVD mortality. At baseline, 256 consecutive patients attending the Kuopio University Hospital coronary angiography unit and with a confirmed diagnosis of CAD were recruited to participate in the KOHH study. Also, 250 age- and sex-matched controls were recruited from the general surgery or otorhinolaryngology departments at the same hospital. The controls were determined by ‘not having heart disease’ based on their medical history and the pre-admission tests. The controls resided in the same geographic area where the cases arose. The same exclusion and inclusion criteria were applied to the control subjects. For further details regarding this cohort, please refer to the previous publications [14,15].

### 2.3. Endpoint Determination

The CVD mortality data were obtained from the Finnish statistics department every year from 2009 to 2015. The current study used the mortality report of 2015. Using the World Health Organization’s International Classification of Diseases-10 codes, I00 through I99 were considered CVD mortality due to atherosclerotic heart disease and stroke. All-cause mortality was compiled by all the deaths occurred in this cohort. The reliability of these data was very high, with 99% after comparing the 2009 and 2011 records in a random sample of 100 records.

### 2.4. Predictor Assessment

The exposure, that is, Oral Hygiene Self-care (OHS), was assessed via questionnaire. Toothbrushing was assessed in 4 categories: (1) brush never or seldom; (2) brush several times a week; (3) brush at least once a day; and (4) brush more than once daily. We created a dichotomy of brushing by combining the lower two and upper two groups. Similarly, flossing was evaluated ((1) never; (2) once a week; (3) several times per week; and (4) daily) and we created dichotomous variable by combining lower two groups and upper two groups. We combined brushing and flossing variables and created 4 mutually exclusive categories: 1. no brushing or flossing, 2. no brushing but flossing, 3. brushing daily but no flossing, 4. both brushing and flossing. Group 2 had only 4 persons who did not brush but flossed. We merged these 4 subjects to the poor OHS group. Thus, we created 3 levels of OHS: the poor group (OHS1) included those who neither brushed nor flossed and 4 individuals who only flossed without brushing; the fair OHS group (OHS2) included those who brushed daily but did not floss; the good oral hygiene group (OHS3) included those who brushed daily and flossed.

Self-reported OHS is potentially unreliable due to reporting bias. Thus, we validated self-reported OHS by clinical markers of oral inflammation, i.e., gingival bleeding index and dental cleanliness, mean plaque index. 

### 2.5. Confounding Factors

Age in years and smoking in three categories (never, past and current smokers) were assessed. Weights were measured without shoes and in light clothing. Heights were measured without shoes using a stadiometer with Frankfort plane in a horizontal position. Body mass index (BMI) was calculated by weight in kg divided by squared height in meters. Hypertension (HTN) and diabetes were ascertained by medical record review. To avoid confounding by affluence and high socioeconomic status, we collected data on educational levels, income and private insurance status. The mean plaque score was assessed by dental examination and calculated by summing each surface plaque score and dividing by the number of surfaces. Gingival bleeding index was calculated by summing all bleeding on probing surfaces and divided by the number of teeth involved. 

### 2.6. Statistical Analyses

Using the Statistical Analysis System (SAS) Version 9.4, we performed chi-square tests, Kruskal–Wallis tests, and proportional hazards regression tests to assess the effect of oral hygiene on all-cause mortality and longevity. The original data from 506 individuals decreased to 476 due to missing values in the predictor, outcome or confounding variables.

The oral hygiene status was categorized into four groups: the edentulous group (OHS0), which included the control, poor OHS group (OHS1), fair OHS (OHS2) group, and the good OHS (OHS3) group. We assessed the basic characteristics, such as age, sex, smoking, number of remaining teeth, and the mean plaque scores among the groups. The categorical variables were compared by the chi-square tests, and continuous variables were tested by non-parametric Kruskal–Wallis test.

Next, multivariable controlled analyses were performed by proportional hazard regression, for adjusting age, sex, smoking in three categories (never, current, and past smokers), body mass index, and education as a socioeconomic status (SES) marker. To test whether OHS is mediated by inflammation, we adjusted for CRP levels. We also tested the interaction of OHS with smoking or dyslipidemia.

## 3. Results

**Figure 1:**
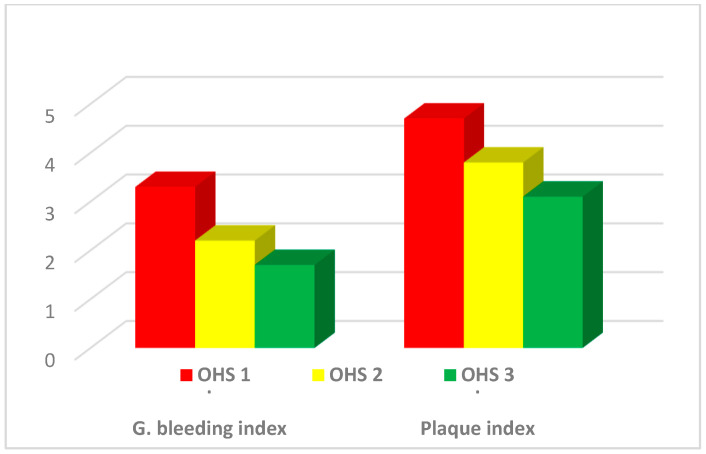
Validation of OHS by gingival bleeding and plaque indices.

By the end of May 2015, 160 all-cause mortality cases had been accrued. The validation of self-reported OHS clearly demonstrated that OHS had an inverse relationship with mean gingival bleeding index (oral inflammation marker) and mean plaque index (dental cleanliness marker), showing good validity in this cohort. The edentulous group could not be assessed for gingival bleeding or plaque indices due to lack of dentition. These results are presented in **Figure 1**.

**Figure 2:**
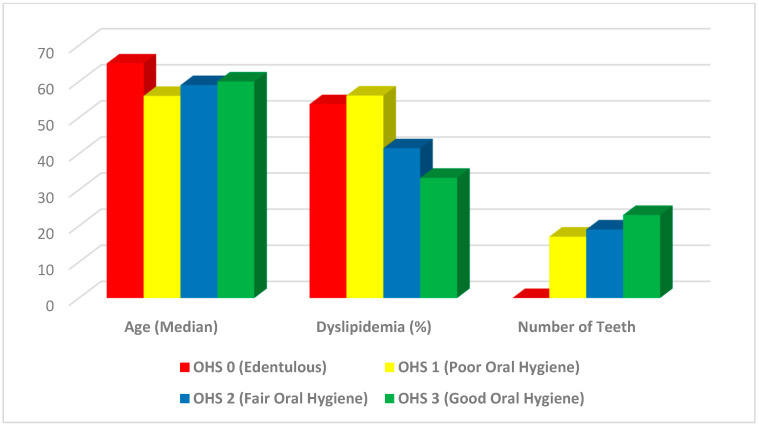
Graphical presentation of selected baseline variables.

**Figure 3:**
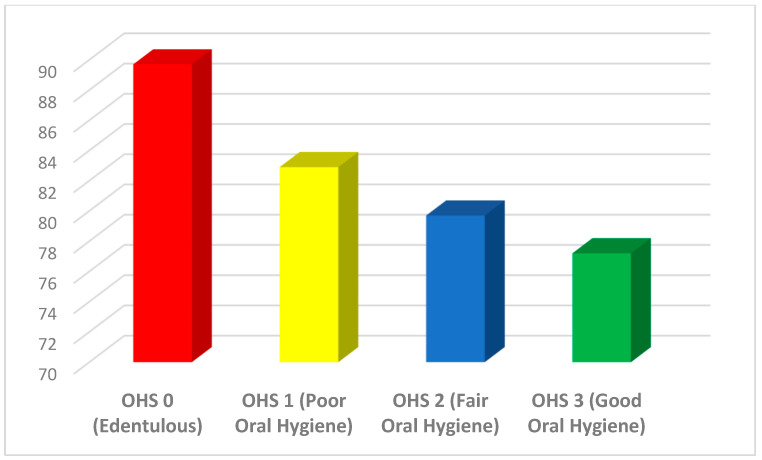
Proportion of CRP > 3 mg/L stratified by oral hygiene self-care.

The baseline characteristics are summarized in **Table 1**, **Figure 2** and **Figure 3**. The control group (OHS 0) included 147 edentulous participants (29.1% of the cohort), the OHS1 group comprised 41 who did not or rarely brushed (8.1%), the OHS2 group consisted of 261 (51.6%) participants who brushed daily but did not floss, and the OHS3 group included 57 (11.3%) subjects who practiced both daily brushing and flossing.

The OHS1 group (rarely or never brushed) tended to be younger, mostly male, and had high prevalences of dyslipidemia and current smoking (Figure 2). The edentulous group was the oldest with a higher prevalence of diabetes than other groups (Table 1, Figure 2).

The BMIs of four groups are all within normal limits in this cohort, which explains the lower rate of diabetes and periodontitis [13]. CRP levels and dental plaque scores were inversely associated with improving OHS, confirming that OHS was associated with decreased systemic and oral inflammation (Figure 1 and Figure 2).

We next calculated the longevity extension from the baseline of each group. The group that brushed and flossed had the highest longevity extension (17.7 years), followed by those who brushed daily or did not brush at all (16.9 years) and the edentulous group (15.2 years). Those who did not brush at all (OHS1) had the same 16.9 years as the daily brushing (OHS2) group, but if we consider the baseline mean age, the OHS1 group had a shorter lifespan of 72.9 years compared to the OHS2 group’s 75.9 years. 

To reflect the time component, we calculated the incidence rate (IR) for each group. The IR of all-cause mortality for the edentulous group (OHS0) was 18.4 deaths per 1000 person-years, the OHS1 group had 11.6 deaths, the OHS2 group had 10.4 deaths, and the OHS3 group had 5 deaths per 1000 person-years, respectively. The IR is an unadjusted assessment but clearly showed that improving OHS had an inverse association with all-cause mortality. To examine how confounding would affect the unadjusted results, we performed multivariate-adjusted analyses.

Multivariate analyses demonstrated that even after controlling for confounders, such as age, smoking, BMI, and education, the good OHS group (OHS3) remained a significant predictor of decreased mortality with HR = 0.50 (0.25–0.99) *p* = 0.045. Poor or fair OHS groups had HRs of 0.95 and 0.88, respectively, and the *p*-values were not significant. These results are presented in Table 2.

The additional adjustment for CRP attenuated the risk reduction slightly (HR = 0.51) and the *p*-value became marginally significant (*p* = 0.06), indicating the OHS impact on all-cause mortality is, at least in part, due to inflammation. This is a simplified mediation analysis and we further explained the principle of mediation analyses in the Discussion. These results are presented in **Table 3**.

When both CRP and dyslipidemia were adjusted, good OHS was significant with HR = 0.51, *p* = 0.05). This is because the poor OHS group (OHS1) had higher dyslipidemia and adjusting for dyslipidemia showed a more salient influence of oral hygiene on all-cause mortality. These results are presented in **Table 4**.

The interactions between smoking and OHS (*p* = 0.53) and dyslipidemia with OHS (*p* = 0.60) were not statistically significant.

**Figure 4:**
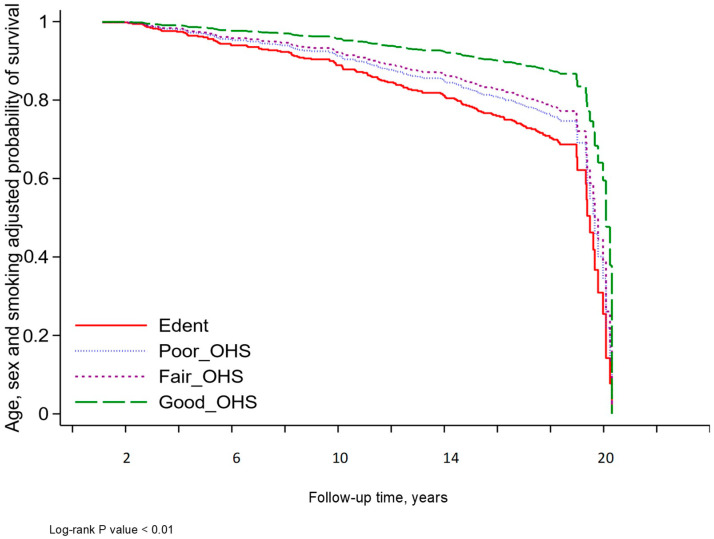
Age-, sex-, and smoking-adjusted survival estimate stratified by oral hygiene self-care.

The survival curve adjusted for age, sex and smoking status and stratified by OHS is presented in **Figure 4**.

## 4. Discussion

We found that good oral hygiene self-care (OHS) was associated with a lower risk for all-cause mortality. Our observation alone may not establish causality. A surprising fact is that those who had 15 teeth or more (OHS1) and did not brush their teeth had similar rates of all-cause mortality as the edentulous group, with HR = 0.98, *p*-value 0.94. This fact suggests that just having more teeth is not sufficient to decrease the risk of mortality. Rather, the dentition should be maintained with good oral hygiene to decrease the risk of all-cause mortality. 

The interpretation of the Hazard ratios (HR) is as follows: HR = 1 denotes that the risk for all-cause mortality between the compared group and the control (edentulous) group is the same. The HR = 0.98 and *p*-value 0.95 for the OHS1 group are very close to 1, and this means the risk of all-cause mortality is very similar to the same in the edentulous group. On the contrary, HR < 1 (for example, HR = 0.5 in the case of the good OHS group (OHS3) implies that the risk of all-cause mortality is reduced to 0.5 of the control (edentulous) group). In other words, the risk of all-cause mortality in the good OHS group is half of the risk in the edentulous group. Another study observed that group brushing 1–2 times showed aOR = 0.26, while brushing after each meal was associated with aOR = 0.65. aOR is a similar measure of HR and both are < 1, which designates that the risk of the outcome is reduced but 0.26 is smaller than 0.65, and this denotes a more robust risk reduction. When HR > 1, the interpretation is straightforward. The higher the HR, the higher risk for the outcome. 

We previously reported that improving OHS was inversely associated with CRP levels in a dose–response manner [2]. This is in agreement with a recent systematic review, which reported that oral hygiene practice was associated with reduced cardiometabolic risk factors and CVD mortality [16]. Taken together, it appears that inexpensive and non-invasive brushing and flossing have the potential to reduce systemic inflammation. This theorem, however, has to be corroborated in randomized controlled trials.

The purpose of adjusting for CRP (Table 3) was to determine whether the pathway OHS influences all-cause mortality was mediated by inflammation reduction. As we reported previously, in mediation analyses [15], the main predictor (OHS) loses significance when the mediator (CRP) is in the model [15]. Therefore, the insignificant *p*-value for OHS (*p* = 0.06) is expected. This result suggests that the pathway linking OHS with all-cause mortality is probably via inflammation.

Additionally, another randomized controlled clinical study corroborated our finding that tooth brushing and flossing significantly reduced interproximal gingival inflammation beyond tooth brushing alone [17]. Thus, these reports underscore the critical role brushing and flossing play in reducing inflammation.

The recent systematic review that assessed the association of OHS with systemic biomarkers of CVD, diabetes mellitus (DM), and chronic kidney disease (CKD) was thorough and clearly laid out the potential associations of relevance [16]. In the review, the majority of the studies reported beneficial effects of OHS on systemic outcomes. One finding that was highlighted in [16] was that “Tooth Brushing (TB) after every meal was more detrimental to kidney function than TB 1–2× daily”, citing the study by Hirano, K et al., 2022 [18]. However, our in-depth assessment of this study [18] revealed “Brushing teeth once to twice a day was associated with an adjusted Odds ratio (aOR) = 0.26 for composite renal outcomes than brushing teeth less frequently, and brushing after each meal was associated with aOR= 0.65”. Thus, both brushing frequencies (1–2/day or 3 times/day) were associated with a lower risk for kidney diseases, albeit brushing 1–2 times/day demonstrated more robust risk reduction than brushing after each meal [18]. We suggest the correct interpretation is that “Tooth brushing after each meal decreased the risk of kidney diseases outcomes by 35% while brushing 1–2 times daily was associated even further risk reduction by 74%”. In essence, both brushing 1–2 times/day and 3 times/day was associated with reduced risk of CKD outcomes. However, 1–2 times daily was associated with more robust risk reduction than brushing 3 times daily.

Additionally, Hirano et al. reported that their findings were observed “only within the low and moderate baseline risk group”, and this is an important point. When the kidney disease is severe, there is overwhelming systemic inflammation. This likely abrogates any beneficial effects that better OHS might have. The same type of observation was reported with the well-designed RCT conducted in type 2 diabetes (T2D) patients by Kaur and colleagues [19]. Although under-powered, the study compared sonic toothbrushing and water rinsing vs. manual toothbrushing with chlorhexidine rinsing. Both groups improved gingival bleeding index, pocket depth, and HbA1c after 4 months. But there were no differences between the compared groups [19]. This, by no means, suggests the futility of OHS. Rather, this trial teaches us that the impact of OHS interventions may be obscured by late-stage diabetes. Alternatively, a study should be conducted among the individuals with prediabetes, not among full-blown T2D patients, to observe significant risk reduction. Further, the sample size should be sufficiently large, and two compared interventions should have a reasonable gradient of efficacy.

Kuwabara and colleagues [20] focused on tooth brushing after each meal as the predictor. They reported that not brushing after each meal was associated with the increased risk of developing diabetes, dyslipidemia, hypertension, and hyperuricemia [20]. These observations are consistent with our results.

In another study by Palmer and coworkers, OHS decreased all-cause mortality but not CVD mortality in end-stage renal disease (ERD) patients [21]. Because ERD patients are immunocompromised and at least 20% die of infections [22], it is plausible that OHS reduced all-cause mortality by reducing infections from oral sources [21]. Reichert and colleagues assessed OHS as the secondary prevention of CVD among coronary heart disease patients [5]. Both studies reported that OHS was associated with a decreased risk of all-cause mortality.

Zhuang et al. reported that poor oral hygiene was associated with a higher risk of major vascular disease, cancer, COPD, liver cirrhosis, and all-cause deaths, but not type 2 diabetes and chronic kidney disease [23].

## 5. Strengths and Limitations

The limitations of our study include the following:

1. Due to the observational study design, residual confounding is possible, although we controlled for the most important confounding factors. Those who practice good oral hygiene may also maintain a healthy lifestyle. However, we adjusted for education, which is a marker for a healthy lifestyle, and OHS still maintained its significance.
2. This study could introduce reporting bias by relying on self-reported OHS. Practically, oral hygiene self-care can only be assessed via self-report. To address this inherent problem, we validated OHS to the clinical measurements of oral inflammation, gingival inflammation, and plaque indices (refer to Methods section).
3. Another limitation is that we did not have data on diet or physical activity. Diet influences mortality via obesity-related inflammation, on CVD, diabetes, and cancer [24]. Given the fact that our cohort has a mean BMI of 23–24 and low prevalence of diabetes, confounding from diet or exercise may be minimal. A recent systematic review [16] reported a similar mortality risk reduction to our results, and we feel that residual confounding due to diet or physical activity in our study may be negligible.
4. This Finnish cohort might have the advantage of high living standards, and generalizability may be limited. However, the systematic review that summarized results from different cohorts with different ethnicities, different living standards, or geographical regions reported similar results [16]. Thus, it appears that the benefits of good oral hygiene may transcend sociocultural and healthcare system differences.

The strengths of our study include a long follow-up period and extensive oral and systemic health data. Thus, we can adjust for most systemic diseases’ impact. For example, some studies suggest that alcohol abuse was a risk factor for all-cause mortality in Finland [25] and we did not have alcohol abuse data. Therefore, we checked the liver cirrhosis mortality, as a proxy for alcohol abuse. There was only one death due to liver cirrhosis in our data. Thus, we are assured that alcohol abuse may not be an important contributor to all-cause mortality in this cohort.

## 6. Conclusions

Our study suggests that good oral hygiene self-care is associated with a lower risk of all-cause mortality. Additionally, among the dentate subjects with more than 15 teeth, if oral hygiene was poor, the mortality risk was similar to edentulism. However, due to its observational study design, our results cannot be inferred as a causal relationship. Future well-designed randomized trials are warranted to establish causality.

## Figures and Tables

**Table 1:** Baseline characteristics stratified by edentulism and oral hygiene self-care.

	Edentulous (N = 147)	Seldom or Not Brushing (N = 41)	Daily Brushing Only (N = 261)	Daily Brushing and Flossing (N = 57)
**Age, Median (IQR)**	65 (59–70)	56 (49–63)	59 (51–65)	60 (52–65)
**Sex, N (%)**				
**Male**	76 (51.7%)	40 (97.6%)	180 (69%)	26 (45.6%)
**Female**	71 (48.3%)	1 (2.4%)	81 (31%)	31 (54.4%)
**Body Mass Index,** **Median (IQR)**	24.3 (22.4–26.3)	25.6 (23.6–28.2)	24.7 (23.0–26.9)	24.5 (23.2–26.0)
**Smoking, N (%)**				
**Never**	93 (66.4%)	17 (42.5%)	179 (69.9%)	42 (76.4%)
**Current**	12 (8.6%)	10 (25%)	24 (9.4%)	4 (7.3%)
**Past**	35 (25%)	13 (32.5%)	53 (20.7%)	9 (16.4%)
**Education (years)**	11 (9–11)	11 (11–11)	12 (11–14)	12 (11–14)
**Diabetes, N (%)**				
**Yes**	24 (17.4%)	5 (12.8%)	19 (7.6%)	2 (3.6%)

Categorical variables are tested by chi-square test. Continuous variables are assessed by Kruskal–Wallis test.

**Table 2:** Multivariable-adjusted proportional hazard model compared with edentulism.

Parameter	*p*-Value	Hazard Ratio (HR)	95% Confidence Limits for HR	
**Poor oral hygiene (OHS1)**	0.88	0.952	0.497	1.825
**Fair oral hygiene (OHS2)**	0.51	0.878	0.594	1.297
**Good oral hygiene (OHS3)**	0.045	0.497	0.251	0.985
**sex**	0.124	0.718	0.471	1.095
**age**	<0.0001	1.106	1.080	1.133
**smoking**	0.0004	1.433	1.176	1.746
**BMI**	0.7045	0.990	0.940	1.042
**Education**	0.5613	0.983	0.926	1.042

BMI: body mass index.

**Table 3:** C-reactive protein added to the basic model in Table 2.

Parameter	*p*-Value	Hazard Ratio	95% Confidence Limits for HR	
**Poor oral hygiene (OHS1)**	0.98	0.990	0.515	1.905
**Fair oral hygiene (OHS2)**	0.63	0.908	0.613	1.345
**Good oral hygiene (OHS3)**	0.058	0.513	0.257	1.021
**sex**	0.1410	0.728	0.476	1.111
**age**	<0.0001	1.109	1.082	1.136
**smoking**	0.0004	1.435	1.173	1.755
**BMI**	0.7042	0.990	0.940	1.043
**Education (in years)**	0.5368	0.981	0.925	1.041
**C-reactive Protein (mg/L)**	0.9141	1.000	0.994	1.007

**Table 4:** C-reactive protein and dyslipidemia added to the basic model.

Parameter	*p*-Value	Hazard Ratio (HR)	95% Confidence Limits for HR	
**Poor oral hygiene**	0.94	0.98	0.51	1.89
**Fair oral hygiene**	0.60	0.90	0.61	1.34
**Good oral hygiene**	0.05	0.51	0.26	1.01
**sex**	0.13	0.72	0.47	1.15
**age**	<0.0001	1.11	1.08	1.14
**smoking**	0.0004	1.44	1.18	1.75
**BMI**	0.73	0.99	0.94	1.04
**Education (years)**	0.52	0.98	0.92	1.04
**Dyslipidemia**	0.74	0.94	0.66	1.34
**C-reactive Protein (mg/L)**	0.88	1.00	0.99	1.00

## Data Availability

This is a private dataset and not available to the public to protect the participants’ privacy. However, limited access may be allowed with the approval of all authors involved in data collection.
